# Discursive constructions of employee mental health in South African labour legislation and organizational policy: implications for psychosocial safety climate

**DOI:** 10.3389/frhs.2026.1879780

**Published:** 2026-09-23

**Authors:** Nthabeleng Innocentia Mdhluli

**Affiliations:** Department of Industrial and Organisational Psychology, University of South Africa, Pretoria, South Africa

**Keywords:** critical discourse analysis, health workforce governance, mental health services, occupational health policy, psychosocial safety climate, reasonable accommodation, South Africa, workforce well-being

## Abstract

South African labour legislation and organizational policies provide fragmented and largely reactive entry points for employee mental health protection, yet the discursive mechanisms through which this pattern is reproduced remain underexamined. This study reports a qualitative documentary analysis of how legislative and organizational policy texts construct employee mental health in South Africa and considers the implications of these constructions for psychosocial safety climate (PSC) and the constitutional rights to dignity, equality, and access to healthcare services. A Fairclough-informed critical discourse analysis was applied to four principal employment statutes, namely the Labour Relations Act, Basic Conditions of Employment Act, Occupational Health and Safety Act and Employment Equity Act, and twelve purposively sampled organizational mental health policies from the public, private and non-profit sectors across five provinces. The analysis was structured by three regulatory dimensions: psychosocial hazard prevention, reasonable accommodation for psychosocial disability and protection from mental health-related discrimination. Although the statutes provide partial and indirect protections, mental health was constructed predominantly through discourses of incapacity, physical safety, and residual disability, with limited textual emphasis on proactive psychosocial risk prevention. The organizational policies largely reproduced these constructions and reflected cultural invisibility and a lack of comprehensive accountability mechanisms. Employees were positioned primarily as recipients of discretionary, individually initiated support rather than as rights-bearers entitled to structural protection. Trade unions and other collective mechanisms were also largely absent from mental health governance. These patterns may constrain the regulatory and organizational conditions associated with a strong PSC, although PSC and employee outcomes were not measured. The study recommends explicit psychosocial risk provisions, strengthened accommodation for psychosocial disability, culturally responsive policy design, preventative human resource practices and the inclusion of psychosocial working conditions within collective bargaining. The findings demonstrate how legislative and policy discourse may shape organizational mental health governance in an upper-middle-income economy.

## 1 Introduction

Psychosocial work conditions are among the most important factors influencing employee mental health, organizational functioning, and workforce sustainability (1, 2). Research consistently links a poor psychosocial safety climate to higher rates of psychological distress, burnout, and work disengagement across national and sectoral contexts (3, 4). Although psychosocial safety climate is conventionally conceptualised at the organizational level, it may also be influenced by the wider legislative and regulatory environment within which organizational policies and practices develop (5). Where those frameworks are silent or fragmented, employers receive a weaker regulatory signal that psychological health constitutes a mandatory governance priority, and employee mental health may be left structurally unprotected.

In South Africa, this legal signal appears ambiguous and inconsistent. The Constitution guarantees dignity (Section 10), equality (Section 9), and access to healthcare services (Section 27). The primary labour legislation, however, does not establish an explicit and integrated framework requiring employers to assess and manage psychosocial risks, operationalise accommodation for psychosocial disabilities, or develop culturally responsive workplace mental health policies. Although existing statutes provide partial entry points for protection, these provisions remain fragmented and predominantly reactive. This gap matters in a context marked by high occupational stress, entrenched socioeconomic inequality, precarious employment, and a culturally diverse workforce for whom stigma and limited access to support services pose additional barriers to protection (6, 7). The costs are significant. Poor workplace mental health has been linked to decreased productivity, absenteeism, higher turnover, and strain on families, communities, and the public health system (8, 9).

The mechanisms through which legislative and policy frameworks influence these outcomes cannot be reduced to the presence or absence of specific provisions. They operate discursively, through the language, assumptions, and silences embedded in legal and organizational texts. These texts construct what mental health is, who bears responsibility for it, and what adequate protection looks like. Critical discourse analysis (CDA) provides a method for investigating these constructions and for examining how they may reproduce or challenge existing power relations (10, 11). This study applies CDA to South African labour legislation and organizational policies. It examines how employee mental health is discursively constructed across three regulatory dimensions, namely psychosocial hazard prevention, reasonable accommodation, and anti-discrimination. It also considers the extent to which these constructions support the conditions associated with a high psychosocial safety climate and the realisation of constitutional rights in the workplace.

### 1.1 Employee mental health in the workplace

Employee mental health has a significant impact on organizational performance and workforce sustainability (2). Poor psychological health at work contributes to reduced productivity, absenteeism, higher turnover, and substantial costs to organizations, families, and the public health system (12). The work-related conditions that damage mental health are well established. Excessive workloads, role ambiguity, job insecurity, inadequate supervisory support, and poor work-life balance are consistently associated with anxiety, depression, and burnout across occupational contexts (1).

South Africa's position as an upper-middle-income country shapes how these risks are produced and governed (13). The country combines a developed constitutional and legislative architecture with severe socioeconomic inequality, uneven occupational health capacity, and unequal access to mental health services. Organizational resources for employee support differ sharply by sector and firm size, and regulatory enforcement capacity is constrained (14). Similar tensions between formal policy ambition and the capacity to manage psychosocial risk in practice are documented across developing and middle-income economies (15). This positioning matters analytically. It explains why legislative text alone cannot be treated as evidence of effective protection, and why the discursive construction of obligations warrants examination in its own right.

Within this setting, socioeconomic conditions exacerbate workplace mental health risks. High unemployment, entrenched inequality, and widespread poverty contribute to psychological distress in the working population (6). Precarious employment, common in mining, agriculture, and the service sector, exposes workers to ongoing uncertainty (8, 16). Structural inequities, including racism and gender discrimination, compound these vulnerabilities and produce disparities in mental health outcomes across the workforce (17, 18). Absenteeism, presenteeism, reduced productivity, and increased turnover intentions are well documented, as are chronic disability and rising healthcare costs at the population level (2, 9, 19, 20).

Despite this evidence, mental health remains insufficiently prioritised in many South African workplaces. Cultural stigma, low mental health literacy, and limited access to support services reduce intervention uptake and contribute to underreporting of psychological distress (7). Many organizations lack comprehensive mental health policies. Where policies do exist, they frequently fail to provide culturally appropriate frameworks for early detection or effective support (2, 21). Addressing these gaps requires strategies that integrate prevention, early intervention, and culturally sensitive support, and that account for both psychosocial risk factors and the broader socioeconomic determinants of psychological health (19).

### 1.2 Legislative and policy frameworks in South Africa

Four statutes form the primary legal framework for employment in South Africa, and each was included in this study's documentary corpus because it governs a distinct dimension of the employment relationship within which psychosocial protection could plausibly be located. The Labour Relations Act 66 of 1995 (LRA) governs collective bargaining, trade union rights, unfair dismissals and labour practices, and dispute resolution through the CCMA and the Labour Court (22). Its incapacity and fair labour practice provisions have a direct bearing on how mental health conditions are managed within the employment relationship. The Basic Conditions of Employment Act 75 of 1997 (BCEA) establishes minimum conditions of employment, including working hours, leave entitlements, and rest periods (22). These conditions shape psychosocial risk exposure even where the Act does not name psychological health. The Occupational Health and Safety Act 85 of 1993 (OHSA) is the primary statute governing employer duties to protect workers from occupational hazards and is the instrument most directly relevant to psychosocial hazard prevention. The Employment Equity Act 55 of 1998 (EEA) prohibits unfair discrimination and provides for affirmative action and the reasonable accommodation of persons with disabilities, whose statutory definition includes mental impairment. None of the four statutes was designed with psychosocial risk governance as a central organising purpose.

The framework is not silent in absolute terms, and this point requires acknowledgement before the analysis proceeds. The BCEA's regulation of working time, rest, and leave may indirectly limit some psychosocial risks (23). The LRA provides procedural fairness in dismissal and establishes collective bargaining machinery through which psychosocial working conditions could, in principle, be negotiated. Section 8(1) of the OHSA imposes a general duty to provide a working environment that is safe and without risk to the health of employees, and its wording does not confine health to the physical dimension. The EEA includes mental impairment within its definition of disability, and the Code of Good Practice on the Employment of Persons with Disabilities offers accommodation guidance. These provisions constitute partial, indirect, and mainly reactive entry points for mental health protection. What the framework does not establish is an explicit and integrated system of psychosocial risk prevention, accountability, and enforcement. That distinction, rather than an absence of any protection, is the position this study examines and defends (2, 24, 25).

Organizational policies occupy the implementation layer between legislation and individual employment relationships. Research in South African workplaces shows substantial variation in how organizations develop and apply mental health policy. Many lack formalised, comprehensive strategies. Where policies exist, they frequently lack culturally sensitive approaches or enforceable mechanisms (2, 21). This inconsistency limits equitable access to mental health support and may hinder the practical realisation of constitutional rights across industries and demographic groups. Coordinated development at both the legislative and organizational levels is therefore widely advocated, with legislation establishing explicit mental health obligations and organizations developing culturally informed policies supported by accountability and monitoring mechanisms (26, 27).

### 1.3 Human rights and mental health

South Africa's Constitution provides the normative foundation for workplace mental health protection. Section 27 guarantees the right of access to healthcare services. Section 10 protects human dignity. Section 9 guarantees equality. These constitutional provisions provide a normative foundation for inclusive, equitable and dignified employment. Although the right of access to healthcare services primarily imposes obligations on the state, constitutional values also inform the interpretation and development of labour legislation and organizational duties relating to employee health, equality and dignity (19, 28, 29). International frameworks reinforce these domestic obligations. The International Labour Organization's occupational health and safety standards acknowledge mental health as an important component of worker well-being and require member countries to implement policies that reduce psychosocial risks (30). Current scholarship treats mental health as a fundamental human right that extends beyond the biomedical model (31, 32), and legislative and organizational responses are increasingly expected to prioritise proactive prevention over reactive remediation (2, 33). This orientation aligns with South Africa's constitutional values of dignity, equality, and social justice, and it establishes a normative basis for holding both legislators and employers accountable for the psychosocial conditions they create and sustain.

### 1.4 Cultural responsiveness in workplace mental health

South Africa's cultural diversity requires mental health policies responsive to the varied experiences and meaning systems of a heterogeneous workforce. Culturally informed interventions improve engagement, trust, and treatment efficacy, particularly in workplaces where stigma and misconceptions about mental illness function as barriers to care (7). Generic workplace mental health models tend to be insufficient in this context. Policies should incorporate culturally appropriate practices that acknowledge employees' cultural backgrounds, belief systems, and community contexts. Existing South African scholarship suggests that cultural considerations are often insufficiently integrated into organizational mental health interventions and policies, which limits their applicability across diverse workforces (34) and narrows the practical reach of constitutional equality guarantees. This study contributes to the scholarship by applying CDA to legislative and organizational policy documents in order to identify the discursive mechanisms through which cultural responsiveness appears to be excluded from workplace mental health frameworks.

### 1.5 Organised labour, collective bargaining and workplace mental health

Organised labour has been central to workplace transformation in South Africa. Trade unions contested managerial authority under apartheid and shaped occupational health, dignity, and worker voice during the democratic transition (35, 36). Collective bargaining has historically addressed wages, working time, safety, and fair treatment. Each of these domains bears directly on psychological health. Workload, bullying, harassment, restructuring, and job insecurity are conditions of work that may legitimately fall within collective consultation and bargaining processes, rather than being treated only as individual clinical concerns. Health and safety committees, which the OHSA requires in prescribed circumstances (ss 19–20), provide an existing statutory structure through which psychosocial risks could, in principle, be raised, discussed, monitored and referred to employers or inspectors for appropriate action.

The capacity of organised labour to carry this agenda is uneven. Recent scholarship documents fragmentation, politicization, and variable institutional capacity, particularly in the public service (37, 38). These constraints qualify, but do not eliminate, the potential of collective mechanisms. They also sharpen the analytical question this study pursues. If collective bargaining has historically shaped South African employment conditions, the visibility or invisibility of trade unions and collective voice within mental health governance texts is itself a significant discursive datum. The analysis therefore examines whether the statutes and organizational policies position organised labour as a participant in psychosocial governance and considers the implications of its presence or absence for collective employee voice.

### 1.6 Psychosocial safety climate as the theoretical framework

Workplace mental health is shaped by more than individual coping resources or job-level demands. It is also influenced by the broader institutional and organizational conditions that govern whether employee psychological health is treated as a governance priority. Psychosocial safety climate (PSC) theory provides the theoretical framework for this study.

PSC refers to shared perceptions of organizational policies, practices and procedures for the protection of worker psychological health and safety (4). It is conceptualised as a precursor to the psychosocial work environment. When PSC is high, organizations invest in preventative practices and allocate adequate resources for psychological health. When PSC is low, psychosocial hazards accumulate and health outcomes deteriorate (3, 4). Empirical evidence shows that PSC is a strong predictor of job demands, psychological distress, burnout, and work disengagement across multiple national and sectoral contexts (3).

Although PSC is conventionally conceptualised at the organizational level, the broader legislative and regulatory environment may shape the conditions under which organizational priorities and practices develop (5). Explicit employer duties, regulatory guidance and enforcement mechanisms can communicate whether psychological health is treated as a mandatory governance concern or a discretionary organizational commitment (5, 30). Where legislation is largely silent on psychosocial risks, employers may receive little regulatory signal that psychological health is a required governance obligation.

This study applies PSC theory to the South African legislative and policy context. It does not measure PSC directly. Instead, it examines how labour legislation and organizational policies construct the priority assigned to employee psychological health. Legislative and policy discourse is therefore treated as a potential macro-level influence on the conditions supporting PSC. The CDA provides interpretive evidence concerning the institutional signals that may shape organizational priorities and practices. The proposed relationship is a discursive pathway rather than a tested causal model.

### 1.7 Conceptual dimensions of workplace mental health regulation

Workplace mental health regulation does not constitute a single, unified legal domain. Three analytically distinct dimensions require differentiation, as each involves different statutory instruments and different legal and organizational responsibilities (2, 25).

The first dimension concerns psychosocial hazard prevention. It addresses work-related risks to psychological health that result from how work is designed, organised, and managed. Excessive workload, role ambiguity, job insecurity, workplace harassment, discrimination and inadequate supervisory support are all potential sources of risk (1, 24). From a psychosocial risk governance perspective, the corresponding organizational responsibility is preventative: psychosocial hazards should be identified, assessed and mitigated before they cause harm. In South Africa, this dimension falls primarily within the scope of the OHSA.

The second dimension concerns reasonable accommodation for psychosocial disability. It applies when an employee has a mental health condition that qualifies as a disability under employment law. The corresponding responsibility is responsive and concerns appropriate adjustments to working conditions, duties or arrangements that support retention or return to work. This dimension is governed by disability and employment equity provisions rather than occupational safety legislation. In South Africa, it falls under the EEA and its associated Code of Good Practice on the Employment of Persons with Disabilities.

The third dimension addresses mental health as a discrimination and equality issue. It concerns situations where employees are treated unfairly or disadvantaged on the basis of their mental health status. Employers are prohibited from discriminating, directly or indirectly, on the basis of disability, including mental health-related disability. This dimension is governed by the EEA's anti-discrimination provisions (Section 6) and the constitutional equality guarantee (Section 9).

The three dimensions are not mutually exclusive. An employee may simultaneously require preventative structural change, reasonable accommodation and protection from discriminatory treatment. Each, however, carries distinct legal obligations and calls for different organizational responses. The Results examine how the four statutes and the sampled organizational policies construct each dimension, and how those constructions align with the constitutional guarantees of dignity, equality and health.

### 1.8 Research questions

This study was guided by three research questions. First, how do South African labour laws and organizational policies discursively construct employee mental health across the regulatory dimensions of hazard prevention, reasonable accommodation, and anti-discrimination? Second, to what extent do these constructions align with the constitutional guarantees of dignity, equality, and health? Third, what are the implications of the identified discursive patterns for psychosocial safety climate and employee well-being in South African workplaces?.

## 2 Materials and methods

### 2.1 Research design and rationale

This article reports a qualitative documentary analysis of four statutes and twelve organizational policies. The documents constitute a primary documentary corpus that was purposively selected, coded, compared, and interpreted. Published scholarship is used to contextualize and interpret the documentary findings rather than constituting the primary analytical corpus.

The analysis is informed by critical discourse analysis (CDA). CDA examines how language in texts reflects, constructs, and sustains power relations, ideologies, and social norms (10, 11). Applied to legal and organizational texts, it reveals the assumptions, silences, and contradictions that underpin how mental health is framed and how employer responsibilities are defined. The analysis was informed by Fairclough's understanding of discourse as operating across textual, institutional and wider social levels (10). At the textual level, attention was given to lexical choice, modality, presupposition, omission and subject positioning. These textual features were then interpreted in relation to the institutional functions of labour legislation and organizational policies and the wider power relations governing workplace mental health (11).

CDA was chosen over thematic analysis, legal doctrinal analysis, and policy content analysis because the research questions demanded attention not only to the content of legislative and organizational texts but also to how language constructs responsibility, legitimacy, institutional silence, and subject positions within workplace mental health governance. The study required a methodology capable of investigating both textual presence and discursive absence, that is, what is said and what is systematically not said. Although thematic analysis could have identified recurring patterns, CDA was better suited to the study's focus on how language constructs institutional responsibility, legitimacy, silence and stakeholder positions. Legal doctrinal analysis would have clarified the formal meaning of the statutes, while policy content analysis would have enabled systematic categorisation of provisions. Neither approach, on its own, would have addressed the interaction between textual choices, institutional power and broader workplace governance as directly as CDA.

### 2.2 Documentary corpus and selection

The documentary corpus comprises two components: four primary statutes and twelve organizational mental health policies. The Constitution of the Republic of South Africa (1996) was not treated as part of the primary analytical corpus. Its guarantees of dignity, equality and access to healthcare services provided the normative framework against which the implications of the legislative and policy constructions were interpreted.

The legislative corpus comprises four principal South African employment statutes selected because of their direct or potential relevance to employee psychological health across the three regulatory dimensions. The LRA was included because it governs the employment relationship, including incapacity, dispute resolution, and fair labour practice obligations. The BCEA was included because it establishes minimum employment conditions, working hours, and rest periods that affect psychosocial risk exposure. The OHSA was included because it is the primary statute governing employer duties to protect workers from occupational hazards and is the instrument most directly relevant to psychosocial hazard prevention. The EEA was included because its anti-discrimination and reasonable accommodation provisions provide the primary statutory framework for mental health-related disability and equality claims. All four Acts were retrieved from the official South African Government website and read in full.

The twelve organizational policies are primary institutional documents, not academic publications. They were purposively sampled from publicly available online sources between January and March 2025. Purposive sampling was selected because the study sought analytical depth and discursive variation rather than statistical representativeness (39). Policies were identified through systematic searches of organizational websites of large South African employers and multinational corporations with significant South African operations, the official websites of national government departments, provincial administrations, and state-owned enterprises, occupational health and human resources practice repositories, and targeted internet searches using term combinations including “mental health policy”, “employee wellness policy”, “employee assistance programme”, “occupational health policy”, “wellbeing policy”, and “South Africa”. Policies were included if they explicitly addressed employee well-being, mental health support, or occupational health and safety; were publicly accessible; were current at the time of collection, having been published or updated since 2020; and came from South African employers or multinationals with significant South African operations. Generic commitment statements without implementation guidance were excluded, as were policies addressing only physical health. Where multiple candidate policies of the same organizational type were identified, selection was guided by variation criteria to maximise sectoral, size-based, and geographic diversity.

The final corpus provided variation across sectors, organizational sizes, geographic locations and analytical policy orientations while remaining sufficiently bounded for close textual analysis. The corpus was not intended to represent the prevalence of particular policy approaches across South African employers. Province was recorded as a descriptive attribute and no provincial comparison was undertaken. An anonymised corpus profile identifying sector, industry, organizational size, geographic base, year and analytical policy orientation is provided in Appendix A. Analytical policy orientation refers to the substantive framing evidenced in the retained coding record; it is not presented as the organization's formal policy title or internal document classification.

Although the policies were publicly accessible, organizational identities were anonymised. The analytical purpose was to identify cross-document discursive patterns rather than to evaluate or rank individual employers. Anonymisation reduced unnecessary reputational attribution while retaining sufficient contextual information for readers to assess the corpus. This approach is consistent with the study's ethics approval, which classified the research as non-human subjects research relying on publicly available documents. A grouped anonymised corpus profile identifying sector, industry, organizational size, geographic base, year and analytical policy orientation is provided in Appendix A.

### 2.3 Analytical procedure

The analysis combined deductive and inductive movements. The three regulatory dimensions defined in Section 1.7, namely psychosocial hazard prevention, reasonable accommodation for psychosocial disability, and protection from mental health-related discrimination, supplied the initial deductive coding structure. Within and across these dimensions, discursive patterns were developed and refined inductively through close reading: four substantive patterns concerning the construction of mental health (medicalization, individualisation, cultural invisibility and weak accountability) and two patterns concerning actor positioning (employer discretion and attenuated collective voice), the latter reported with the subject-position analysis in the Results. The regulatory dimensions and the discursive patterns therefore occupy different analytical levels. The former structured what was examined; the latter emerged from how the texts were found to construct it.

The first phase involved intensive reading of all selected texts to identify language use related to mental health, employer responsibilities, and cultural sensitivity. The linguistic analysis examined modality, presupposition, lexical choice, omission, and subject positioning to determine how mental health is constructed and positioned in legislative and organizational discourse. NVivo qualitative data analysis software was used to organise and manage the emerging categories. Coding procedures served as organizational tools within the broader interpretive logic of CDA rather than as a standalone thematic method. Coding was iterative, using constant comparison to refine categories and surface finer patterns across statutes, sectors, organizational sizes, and analytical policy orientations, as summarised in the grouped corpus profile in Appendix A. Passages containing both protective and restrictive language were treated as evidence of discursive tension rather than assigned to a single category, and interpretive decisions of this kind were documented in reflexive analytical memos, which also recorded researcher positionality throughout.

Because CDA does not aim for positivist coding reliability, the peer review of the analysis focused on interpretive scrutiny rather than statistical inter-coder agreement. Coding categories and emergent analytical claims were reviewed iteratively by a colleague with expertise in qualitative organizational research. The review considered whether the textual evidence supported the interpretation, whether plausible alternative readings had been considered, and whether the movement from codes to broader discursive claims was coherent. A detailed audit trail was maintained, comprising annotated source texts, dated coding logs, reflexive memos and decision records for category revisions. Requests for access to de-identified components of the audit trail may be considered by the corresponding author, subject to availability and institutional and ethical requirements.

### 2.4 Illustrative coding matrix

Table 1 illustrates the analytical movement from selected documentary wording to broader discursive claims. The legislative extracts are reproduced from the relevant statutes. The organizational rows use exact short lexical expressions retained in the analytical record and reported in Appendix A. No extended organizational-policy quotation has been reconstructed. Table 2 in the Results provides the corresponding organizational-policy code-to-claim pathway.

**Table 1:** Illustrative coding matrix showing the analytical progression from documentary wording to discursive claims.

Documentary wording	Regulatory dimension	Linguistic feature	Initial interpretation	Discursive category	Broader claim
“a working environment that is safe and without risk to the health of employees” (OHSA, s 8 (1))	Psychosocial hazard prevention	Broad, undifferentiated lexical scope of “health”; psychosocial hazards not expressly specified in the Act's definitions or regulations	The general health duty is textually broad, but psychosocial hazards are not expressly specified or operationalised within the primary statute	Physical–technical framing of risk	Psychosocial hazards appear to lack an explicit regulatory home within the primary statute, which may signal discretionary rather than mandatory governance
“a long-term or recurring physical or mental impairment which substantially limits their prospects of entry into, or advancement in, employment” (EEA, s 1)	Reasonable accommodation for psychosocial disability	Definitional inclusion of “mental impairment”; limited specificity in operational provisions and reporting categories for psychosocial disability	Mental impairment is acknowledged in the definition but receives limited operational elaboration	Residual disability framing	The definitional inclusion of mental impairment does not, by itself, establish a proactive system for identifying and addressing psychosocial disability before individual disclosure or accommodation requests arise
“fitness for duty” (mining group, Appendix A)	Psychosocial hazard prevention	Nominalization; the employee is constructed as an operational risk object	Mental health is assessed against operational continuity rather than employee well-being	Medicalised incapacity framing	The policy wording is consistent with a medicalised and operational framing of employee mental health
“resilience”; “performance optimisation” (financial services group, Appendix A)	Psychosocial hazard prevention	Lexical choices assigning capacity and responsibility to the individual employee	Mental health is constructed as a manageable individual capacity	Individual responsibilisation	The lexical emphasis appears to locate responsibility primarily within the individual employee rather than the organization
“incapacity management”; “return-to-work” (public sector group, Appendix A)	Reasonable accommodation for psychosocial disability	Bureaucratic proceduralism; the employee is positioned as an administrative case	Mental health is processed after breakdown rather than prevented	Reactive administrative framing	The wording is consistent with a reactive, procedural orientation towards employee mental health in the state's own employment practice

**Table 2:** Retained organizational-policy lexical evidence and CDA interpretation.

Policy group	Exact retained lexical expressions	Linguistic feature	Subject position	Interpretive category
Mining	“fitness for duty”; “risk management”	Nominalization and technical risk vocabulary	Employee as an object assessed against safe operation and continuity	Medicalised and operational framing
Financial services	“resilience”; “performance optimisation”	Abstract nouns locating capacity and improvement within the individual	Employee as a self-managing performer	Individualisation and responsibilisation
Technology	“wellness days”; “mental health stipends”	Benefits and consumption vocabulary	Employee as an individual user of allocated support resources	Benefits-based individual support
Public sector	“incapacity management”; “return-to-work”	Bureaucratic process nouns and post-harm sequencing	Employee as an administrative case requiring processing	Reactive medical-administrative framing
Non-profit	“burnout prevention”; “compassionate leave”; “staff well-being”	Protective and care-oriented vocabulary without retained procedural detail	Employee as recipient of organizational concern	Aspirational well-being framing

### 2.5 Ethical considerations

Ethical compliance followed institutional and national research ethics guidelines. The Research Ethics Committee of the University of South Africa approved the study (Ref: 8589). The study was classified as non-human subjects research because it relied entirely on publicly available legislative and organizational policy documents and involved no direct interaction with human participants. Organizational policies accessed online were handled with care to protect identifiable organizational information, and no personal or sensitive data were collected or analysed. Appendix A presents anonymised characterisations of the twelve policies, identifying them by sector, size, analytical policy orientation, and primary geographic base rather than by organization name. These measures reflect the principles of confidentiality, respect for organizational autonomy, and responsible research conduct.

## 3 Results

This section presents the findings of the critical discourse analysis across two domains: the four legislative instruments and the twelve organizational mental health policies. For each statute, the analysis first identifies the protection the instrument already provides, then examines how mental health is discursively constructed, what remains implicit, reactive or absent, and the subject positions the text produces. The organizational policy analysis then reports how mental health is constructed across the sampled policies, organised thematically. Cross-cutting discursive patterns that emerged inductively are reported separately from the deductive regulatory dimensions that structured the coding. Interpretation of these findings, including their alignment with prior scholarship and their implications for psychosocial safety climate, constitutional rights and reform, is reserved for the Discussion.

### 3.1 Legislative constructions of employee mental health

#### 3.1.1 Labour relations act

The Labour Relations Act 66 of 1995 is South Africa's primary legislation governing fair labour practices, collective bargaining and dispute resolution. Its mandate emphasises employee rights, including dignity and fair treatment in the workplace (25, 40). The Act provides real, although indirect, protection relevant to mental health. Its procedural fairness requirements constrain arbitrary dismissal, its unfair labour practice provisions offer a route of challenge for employees treated unfairly, and its collective bargaining machinery could, in principle, carry psychosocial working conditions into negotiated agreements.

The Act's direct engagement with mental health is, however, narrow. Mental health considerations appear primarily in the provisions governing incapacity (Schedule 8, Item 10) and the procedural requirements of fair employment practice. These provisions are concerned with assessing an employee's capacity to perform work, considering possible adaptation or alternative employment, and determining whether continued employment is reasonably feasible. They are activated after incapacity has arisen rather than through prior identification of psychosocial risks.

A reactive orientation therefore characterises the Act's discursive construction of mental health. The LRA requires procedural fairness and post-incident dispute resolution, but not proactive measures such as psychosocial risk assessments (25, 33). Across the three regulatory dimensions, the LRA is most relevant to reactive employment protection and the management of incapacity. Its contribution to psychosocial hazard prevention is indirect, while discrimination and reasonable accommodation are addressed more explicitly through the EEA. The subject position produced is that of the employer as procedural compliance actor and the employee as an incapacitated worker requiring procedural management.

#### 3.1.2 Basic conditions of employment act

The Basic Conditions of Employment Act 75 of 1997 establishes minimum employment conditions, including working hours, leave entitlements and rest periods. These provisions constitute a genuine, if indirect, form of psychosocial protection. Limits on working time, guaranteed rest and leave entitlements may reduce exposure to some recognised psychosocial risk factors, particularly excessive workload and inadequate recovery (23).

The Act's protective orientation is directed principally towards quantifiable minimum conditions of employment, including working time, rest and leave. The Act does not frame working time, rest or leave expressly as psychosocial risk controls, nor does it require employers to assess how work organization may affect psychological health. Its contribution to hazard prevention is therefore indirect rather than absent. The subject position produced is that of the employer as provider of minimum conditions and the employee as a worker entitled to measured hours and rest, rather than a psychosocial subject whose health is shaped by how work is organised and managed.

#### 3.1.3 Occupational health and safety act

The Occupational Health and Safety Act 85 of 1993 is the primary statute governing employer duties to protect workers from occupational hazards. Section 8(1) requires employers to provide and maintain, as far as is reasonably practicable, a working environment that is safe and without risk to the health of employees. Read in isolation, this general duty is broad enough to encompass psychological risk, and it constitutes the Act's most significant potential entry point for psychosocial protection. Section 17 provides for health and safety representatives, and sections 19 and 20 provide for health and safety committees, creating participatory structures through which workplace risks can be raised, discussed and referred for action.

The text of the Act does not, however, expressly define, regulate or operationalise psychosocial hazards in the way that it addresses many physical and technical hazards. The Act's regulatory vocabulary of hazardous substances, machinery, structural conditions and physical agents positions the worker as a body at risk of physical injury rather than as a psychosocial subject. Workplace stressors well documented in the South African literature, including harassment, excessive workload, job insecurity and role ambiguity, have a limited regulatory home within the Act's protective architecture (1, 24). Psychosocial risk management is therefore not textually established as an explicit statutory requirement within the primary Act.

Although implementation matters were not part of the primary statutory corpus, existing regulatory and occupational health literature indicates that psychosocial hazards have received limited operational attention in inspection, guidance and training, that no OHSA regulations address the identification, assessment or management of psychosocial risks, and that South Africa has neither adopted the ISO 45003 standard for psychological health and safety management nor issued comparable departmental guidance (41). This contextual evidence is consistent with, and reinforces, the textual finding above; its interpretive significance is examined in the Discussion.

#### 3.1.4 Employment equity act

The Employment Equity Act 55 of 1998 is the most direct statutory basis for mental health protection among the instruments examined. Section 6(1) prohibits unfair discrimination in any employment policy or practice, including on the ground of disability. Section 6(3) addresses harassment as a form of unfair discrimination. Section 15(2)(c) requires designated employers to make reasonable accommodation for people from designated groups, including persons with disabilities. Section 1 defines disability as a long-term or recurring physical or mental impairment which substantially limits prospects of entry into, or advancement in, employment, a definition that expressly includes mental impairment. The Code of Good Practice on the Employment of Persons with Disabilities elaborates a detailed reasonable accommodation framework. Within the accommodation and anti-discrimination dimensions, the EEA therefore provides the strongest formal coverage in the corpus.

Despite this formal scope, the CDA reveals a disparity between the Act's stated coverage and its discursive elaboration of mental health. Mental impairment is a residual category in the EEA's discursive landscape. It is acknowledged in the definitions but receives limited elaboration in the operational provisions, guidance documents and reporting requirements that shape how the Act functions. The Code of Good Practice provides detailed accommodation guidance for employees with disabilities but gives limited explicit attention to the relationship between accommodation and the organizational conditions that may have contributed to psychological harm. This construction is consistent with the reactive, individualised framing identified in the LRA, BCEA and OHSA analyses.

Three features of the textual framework deserve attention. First, the framework does not establish an employer-led mechanism for identifying accommodation needs before an employee discloses a qualifying impairment or requests support. Second, mental impairment is included within the broader statutory disability category, but psychosocial disability is not separately specified in the employment equity planning and reporting architecture, so mental health remains largely unspecified within the Act's equity accountability mechanisms. Third, employees alleging discrimination on the basis of mental health status must show that the condition qualifies as a disability under section 1, a threshold that may be unclear for conditions that are episodic, work-related or not yet formally diagnosed. The implications of these features, including the role of stigma in disclosure and the case for reform, are taken up in the Discussion and Recommendations.

### 3.2 Organizational policy constructions

The following analysis reports how mental health is constructed across the twelve sampled organizational policies. The findings are organised thematically because the sample was selected for discursive variation rather than comparative sectoral inference. Sector, organizational size and analytical policy orientation are used as descriptors within each theme; the geographic distribution of the corpus is reported descriptively in Appendix A, and the corpus does not support comparative claims between provinces, so none are made.

The organizational-policy findings are supported by exact short lexical expressions retained in the analytical record. Extended clauses are not reproduced because a reliable de-identified record of the surrounding wording was not available during revision. No quotation has therefore been reconstructed from memory. Table 2 presents the retained textual evidence and the interpretive movement from wording to discursive claim.

#### 3.2.1 Sectoral vocabularies and framing patterns

The retained lexical evidence shows sector-specific vocabularies but overlapping orientations. In the mining group, “fitness for duty” and “risk management” frame mental health through operational safety and continuity. In the financial-services group, “resilience” and “performance optimisation” construct psychological health as an individual capacity linked to performance. The technology document uses the benefits language of “wellness days” and “mental health stipends”, positioning support as a resource accessed by the individual employee. Public-sector documents use the administrative vocabulary of “incapacity management” and “return-to-work”, making mental health most visible after impairment has affected work participation. The non-profit document uses the more protective vocabulary of “burnout prevention”, “compassionate leave” and “staff well-being”, although the retained record did not contain corresponding procedural detail.

#### 3.2.2 EAP-centred and individual support models

Large organizations in the sample (*n* = 8) consistently maintained formal mental health policies, with Employee Assistance Programmes serving as the primary intervention model. In these texts, mental health is acknowledged, but its management is delegated to external clinical providers and framed as an individual obligation to seek help. Responsibility for the psychosocial conditions associated with distress, such as workload, precarious employment and inadequate support, remains comparatively unspecified.

The three medium-sized documents combined EAP-related support with broader employee-wellness provisions. Because the retained record does not support a reliable document-by-document formal policy classification, no finer distribution is reported. These policies contained less procedural detail than the documents of larger organizations. The single small organization policy contained a brief commitment to “staff well-being” with no implementation mechanisms. The single non-profit policy reflected a broader well-being orientation, citing “burnout prevention” and “compassionate leave” as organizational commitments, but contained no implementation guidance, remaining aspirational rather than operational.

#### 3.2.3 Incapacity, accommodation and return-to-work procedures

Public sector policies (*n* = 4) incorporated mental health into occupational health frameworks and bureaucratic incapacity procedures, consistent with the administrative vocabulary reported in Table 2. None of the four public sector policies referred to the constitutional rights to health or dignity. This textual absence is reported here as a documentary finding; its interpretive significance, given the state's position as employer and constitutional duty bearer, is examined in the Discussion.

#### 3.2.4 Cultural responsiveness and accountability

Two of the twelve policies mentioned multilingual resources. None mentioned traditional healing practices or community-based support systems. One policy referred to culturally appropriate support without defining the term or indicating how it would be operationalised. Across the corpus, employees were addressed through a universal category that did not differentiate cultural or linguistic background.

None of the sampled policies established a comprehensive accountability framework combining measurable indicators, specified monitoring responsibilities, reporting intervals and consequences for non-implementation.

Commitments to mental health across the corpus therefore remained largely aspirational rather than actionable, stated as intentions without institutional mechanisms for delivery.

### 3.3 Cross-cutting discursive patterns

Four cross-cutting discursive patterns emerged across the regulatory dimensions: medicalization, individualisation, cultural invisibility and weak accountability. Two related patterns concerning actor positioning, employer discretion and attenuated collective voice, are addressed separately in Section 3.4 because they concern stakeholder agency rather than the substantive construction of mental health.

The first pattern is medicalization. Across the statutes and the majority of the policies, mental health becomes textually visible primarily as diagnosed incapacity: a certified condition affecting an individual's ability to work. The LRA's incapacity provisions, the public sector policies' “incapacity management” and “return-to-work” protocols, and the mining sector's “fitness for duty” framing share this construction. Mental health enters the text after breakdown, through a clinical or administrative gateway, rather than as an ongoing feature of how work is organised.

The second pattern is individualisation. Responsibility for psychological health is repeatedly assigned to the individual employee. The EAP model delegates management to external providers and positions the employee as a help-seeker; the language of “resilience” and “performance optimisation” constructs mental health as an individual capacity to be developed; and the EEA's textual framework engages accommodation only once an employee discloses or requests support. Organizational and statutory responsibility for the conditions associated with distress is correspondingly muted.

The third pattern is cultural invisibility, evidenced by the findings reported in Section 3.2.4: two of twelve policies mentioning multilingual resources, no reference to traditional healing or community-based support, and a universal employee category throughout. The legislative texts contain no cultural competence provisions, so the organizational pattern extends a silence already present in the statutes.

The fourth pattern is weak accountability, evidenced in Section 3.2.4. The organizational texts extend the legislative pattern: statutes that do not require psychosocial risk governance are mirrored by policies that state commitments without mechanisms. Taken together, the four patterns are mutually reinforcing, and the organizational policies largely reproduce the individualised, medicalised framing of the legislative texts. The relationship of these patterns to prior scholarship on corporate well-being discourse, responsibilisation and symbolic policy adoption is developed in the Discussion.

### 3.4 Subject positions and stakeholder voice

Across the legislative instruments and organizational policies examined, a consistent pattern of subject positioning emerged. Active agency is concentrated in employers and the state, while employees are positioned predominantly as objects of discretionary action rather than as bearers of enforceable psychosocial rights.

Employers are positioned in the four statutes as regulated parties in respect of physical safety but primarily as discretionary actors in respect of psychological health. Under the OHSA, the employer is a technical safety manager with obligations directed at the physical working environment. Under the LRA, the employer is a procedural compliance actor whose incapacity procedures are engaged by employee breakdown. Under the BCEA, the employer is a provider of minimum conditions. Under the EEA, the employer is an equity planner whose accommodation duties are engaged through disclosure or request. None of the four instruments positions the employer as a proactive architect of psychosocial safety. Employer responsibility is therefore constructed as reactive and bounded rather than as an affirmative responsibility to identify and address psychosocial hazards before harm occurs.

Employees are depicted across most of the texts as objects of management action: incapacitated workers requiring procedural management (LRA), workers entitled to minimum conditions (BCEA), bodies at risk of physical harm (OHSA), and members of designated groups whose protection is engaged through disclosure (EEA). In the organizational policies, employees appear as individuals who must seek help, self-identify distress and request assistance. The employee's textual agency is largely confined to help-seeking, and employees are rarely positioned as participants in the governance of the psychosocial conditions that shape their own health.

Trade unions are largely absent from the mental health discourse of the texts examined. The LRA places trade unions at the centre of labour relations generally, yet neither the LRA nor any other instrument in the corpus positions unions as stakeholders in workplace mental health governance, and none of the twelve organizational policies assigns them a role. Health and safety committees, where mentioned, are not connected to psychosocial risk in any of the texts. This absence leaves collective bargaining largely unused as a means of advancing psychosocial risk management. Its historical and institutional significance is developed in the Discussion.

The state occupies a dual position in the corpus: as regulator, it authors the legislative texts analysed; as employer, its four public sector policies contain no reference to the constitutional rights to health or dignity. EAP and healthcare providers occupy an outsourced support position: in the policies that adopt the EAP model, clinical providers external to the organization are positioned as the primary locus of mental health management. This positioning allows organizational texts to acknowledge mental health support while leaving responsibility for the psychosocial conditions associated with distress comparatively unspecified.

Finally, the analysed texts rarely differentiated employees according to employment precarity, cultural or linguistic background, geographic access to services or mental health literacy. Most relied on a universal employee category that did not acknowledge differential exposure to psychosocial risk or unequal access to support. Taken together, these subject positions form a consistent textual architecture: mental health governance rests largely with employer discretion, mental health distress is positioned as the individual employee's concern, collective voice is attenuated, and employee difference is undifferentiated. The interpretive significance of this architecture, including whose interests it may serve, its relationship to constitutional guarantees and its implications for psychosocial safety climate, is developed in the Discussion.

## 4 Discussion

The discussion moves from description to interpretation. It examines what the patterns reported in the Results suggest about the structural conditions governing workplace mental health in South Africa, organised around the psychosocial safety climate framework introduced in Section 1.6. The findings indicate that the limitations of workplace mental health protection in South Africa are not simply a matter of legislative gaps. They reflect a textual architecture that recurs across legislative texts and organizational policies. Figure 1 presents this interpretation schematically.

**Figure 1:**
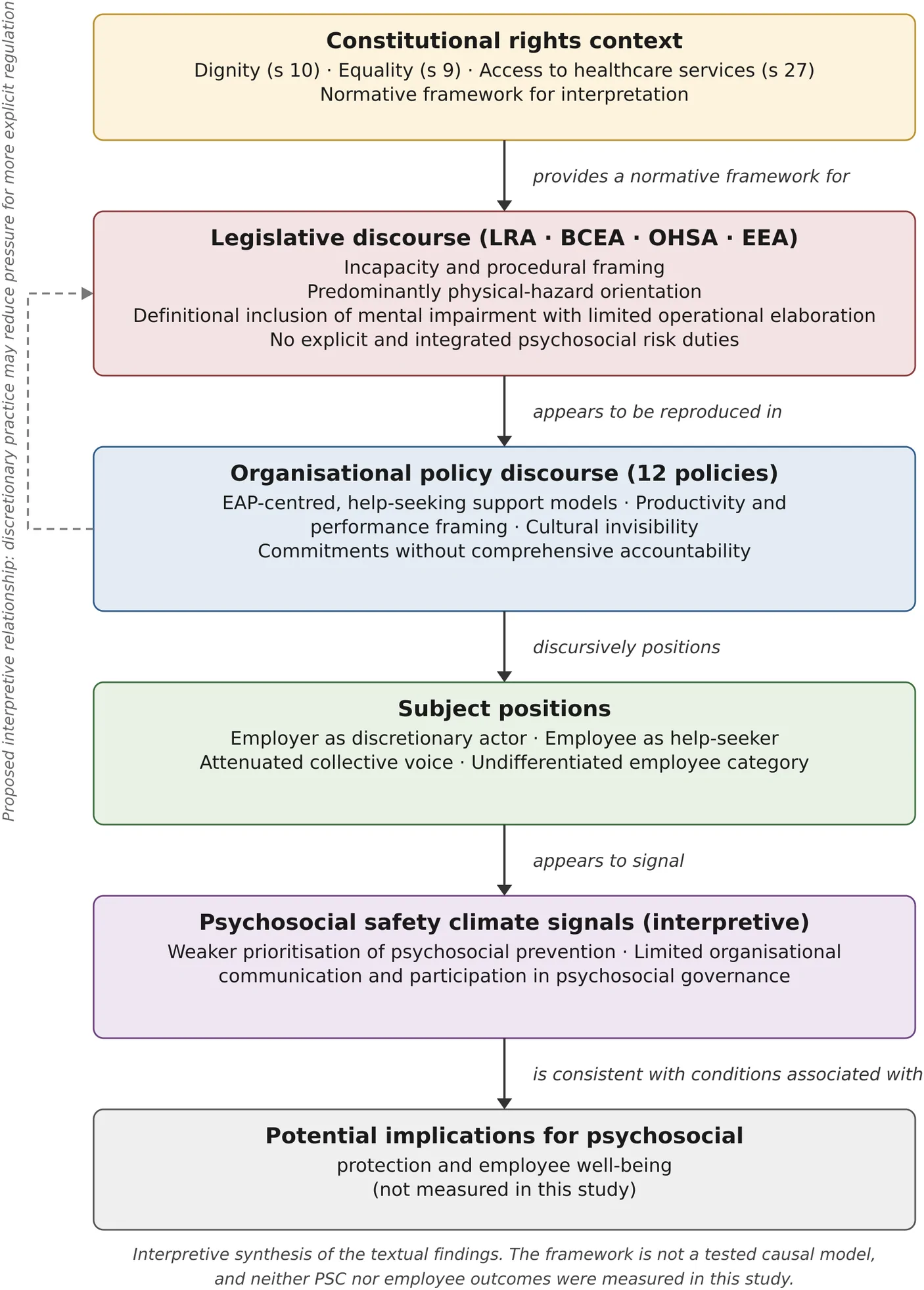
Interpretive framework linking legislative and organizational discourse to psychosocial safety climate signals. The diagram is an interpretive synthesis of the textual findings, not a tested causal model. Arrows represent discursive relationships identified across the analysed texts and are labelled with relational language (may shape, appears to signal, is consistent with). The framework moves from the constitutional rights context through legislative discourse, organizational policy and subject positions to potential implications for psychosocial protection and employee well-being. Neither psychosocial safety climate nor employee outcomes were measured in this study.

### 4.1 Legislative and policy discourse as psychosocial safety climate signals

A consistent pattern emerged across the four legislative instruments. Mental health is constructed primarily as a problem to be addressed after harm has occurred rather than a condition to be prevented through proactive governance. In the LRA, mental health appears through incapacity procedure. In the BCEA, it is peripheral to a framework of quantifiable minimum conditions. In the OHSA, it is not expressly specified or operationalised within a regulatory architecture directed at physical hazards. In the EEA, it is acknowledged in the disability definition but receives limited operational elaboration.

PSC theory offers a productive lens for interpreting this pattern. PSC comprises four interrelated domains: management commitment to psychological health, the priority assigned to psychological health relative to productivity demands, organizational communication about psychosocial matters, and employee participation in psychosocial governance (3, 4). The discursive patterns identified in this study can be read against each domain. Legislation that engages mental health only as certified incapacity appears to communicate weak regulatory prioritisation of prevention, which may constrain the conditions supporting management commitment. Policies that route support through employee-initiated EAP channels may limit organizational communication and participation by positioning employees primarily as help-seekers rather than contributors to psychosocial governance. The near absence of collective voice suggests limited structural participation. These readings identify a potential discursive pathway through which the conditions supporting PSC may be weakened. They do not establish causation. PSC was not measured in this study, at either the organizational or the national level, and the claims advanced are interpretive.

Read in this way, legislative silence on psychosocial hazards such as workplace stress, harassment, bullying and discrimination is not analytically neutral. It leaves proactive employer responsibility textually unestablished and keeps mental health outside the register of collective labour rights. The constitutional guarantees of dignity, equality and health (Sections 9, 10 and 27) risk remaining abstract entitlements where the legislative framework does not require the psychosocial risk assessment, accommodation and culturally competent practice that would translate rights into workplace experience (29, 42).

### 4.2 Discursive reproduction across law and organizational policy

The organizational policies in the sample did not merely operate in the absence of legislative guidance. Their language reproduced and extended the patterns embedded in the law. The productivity and performance framing reported in Section 3.2.1 is consistent with international research on corporate well-being discourse (43, 44). The delegation of mental health management to individual employees, visible in the lexicon of “resilience” and “performance optimisation”, reflects what Cederström and Spicer describe as the responsibilisation of health: organizations acknowledge mental health while assigning its management to the individual (45). The EAP-centred model reported in Section 3.2.2 operates as what critical occupational health scholarship terms a responsibility technology: mental health is recognised, but its governance is outsourced to external clinical providers and the employee's recourse is framed as an individual decision to seek help rather than a collective entitlement to structural protection (1, 46, 47).

The accountability findings reported in Section 3.2.4 are consistent with research on symbolic policy adoption, in which stated commitments are decoupled from institutional mechanisms for delivery (48). Where commitments are aspirational and unmonitored, policy discourse may serve a legitimising more than a protective function, a pattern PSC research associates with the decoupling of stated management priorities from practice (4). The cultural invisibility findings align with criticism of universalist mental health models that overlook indigenous knowledge systems and with evidence that culturally informed interventions improve engagement and efficacy in South African workplaces (7, 21, 27, 49). The aspirational character of the non-profit policy and the limited procedural detail of the medium and small organization policies are consistent with documented capacity constraints in resource-limited organizations and small and medium-sized enterprises (14, 50–52). Disclosure-triggered accommodation, reported for the EEA in Section 3.1.4, carries particular weight in workplaces where mental health stigma is pervasive: disclosure carries reputational risk, and employees who fear adverse consequences may underutilize protections nominally available to them (7, 20).

### 4.3 Convergence across the policy corpus

Variation across sectors and organizational sizes highlights rather than obscures the cross-cutting findings. Sectoral differences in vocabulary were real but convergent: the mining, financial, technology, public and non-profit documents produced overlapping constructions centred on individual support and reactive management. The size-based comparison is significant for PSC theory. Similar individualising constructions occurred across organizations of different sizes, suggesting that resource availability alone may not explain the patterns observed in this corpus. Larger organizations generally possessed more formalised policies and support structures, but greater policy sophistication did not necessarily correspond with a stronger preventative orientation. The findings therefore suggest that organizational capacity and regulatory discourse may operate together, rather than indicating that either factor alone accounts for the observed pattern.

### 4.4 Constitutional alignment and subject positioning

Taken together, the findings indicate a tension between South Africa's constitutional rights framework and the discursive construction of workplace mental health observed in this corpus. The Constitution guarantees dignity, equality and access to healthcare services (Sections 10, 9 and 27). Across the texts studied, mental health is framed primarily as individual incapacity, cultural diversity is largely unaddressed, and proactive employer accountability is textually unestablished. Within the analysed corpus, constitutional rights were seldom translated into explicit organizational responsibilities, implementation procedures or accountability mechanisms.

CDA illuminates how this tension is sustained through language. Through silences, medicalised frames, individualising logics and cultural erasure, the analysed discourse positions employers as discretionary gatekeepers and employees as applicants for assistance rather than rights-bearers entitled to structural protection (53). The state's dual position is particularly revealing: as regulator it authors the legislative discourse, while in the four public-sector policies examined, the state's constitutional commitments were not explicitly connected to workplace mental health responsibilities, revealing a discursive tension within this limited policy sample (54). The undifferentiated employee category reported in Section 3.4 compounds the tension. By treating all employees as equally situated, the texts obscure the differential vulnerabilities produced by South Africa's history of structural inequality, which the equality guarantee is specifically intended to mitigate. Workers whose frameworks for understanding mental health differ from the Western biomedical model embedded in these texts may in practice be distanced from protections the texts purport to provide. Following Fairclough (10) and Wodak and Meyer (11), this architecture is read not as a passive reflection of existing power relations but as a textual practice that may contribute to their reproduction. The findings suggest a potentially reinforcing relationship. Limited legislative specificity may leave organizational responsibilities underdefined, while organizational policies that rely on discretionary and individual support may reduce pressure for stronger regulatory articulation. This proposed relationship remains interpretive and requires empirical examination.

### 4.5 Organised labour and collective voice

The near absence of trade unions and collective mechanisms from the analysed mental health discourse acquires its full significance against the historical role of organised labour in South Africa. Trade unions contested managerial authority under apartheid and shaped occupational health, dignity and worker voice during the democratic transition (35, 36). Workload, harassment, restructuring and job insecurity, the very conditions associated with psychosocial risk, are conditions of work that may legitimately fall within collective consultation and bargaining. Yet none of the twelve organizational policies, and none of the mental-health-relevant provisions identified in the four statutes, assigned unions an explicit role in psychosocial governance, and health and safety committees were nowhere connected to psychosocial risk in the analysed texts.

This absence matters for two reasons. First, it leaves collective bargaining largely unused as a mechanism through which psychosocial protections could be negotiated, monitored and enforced, individualising a domain of working life that organised labour has historically shaped collectively. Second, it removes a counterweight in precisely the situations where individual employees are weakest: where stigma, power imbalances and limited legal literacy make individual assertion of rights unlikely. The capacity of organised labour to carry this agenda is admittedly uneven. Contemporary scholarship documents fragmentation, politicization, and variable institutional capacity, particularly in the public service (37, 38). These constraints qualify, but do not eliminate, the potential of collective mechanisms. Repositioning psychological health as a legitimate subject of collective bargaining would move it from discretionary benefit towards negotiated obligation. Such a shift is conceptually significant and may be pursued through existing collective bargaining, consultation and health and safety structures, although its practical feasibility is likely to vary by sector, union capacity and workplace context.

### 4.6 Comparative perspective

Several jurisdictions provide explicit reference points for psychosocial risk governance. In Australia, the model Work Health and Safety framework addresses psychosocial hazards expressly, supported by a Model Code of Practice that guides duty holders in eliminating or minimising psychosocial risks so far as is reasonably practicable; the legal effect of the model provisions depends on adoption within individual jurisdictions (55). In the United Kingdom, employers have a legal duty to assess and manage work-related stress, while the Health and Safety Executive's Management Standards provide a structured approach covering demands, control, support, relationships, role and change (56). At European Union level, Directive 89/391/EEC establishes general prevention, information, consultation and worker-participation duties, while the autonomous 2004 Framework Agreement on Work-Related Stress provides employers, workers and their representatives with a framework for identifying, preventing and managing work-related stress (57). Mexico's NOM-035-STPS-2018 offers a middle-income example by requiring the identification, analysis and prevention of psychosocial risk factors and the promotion of a favourable organizational environment (58). These comparisons demonstrate that explicit recognition is feasible across different legal and economic settings, while also indicating that regulatory recognition must be accompanied by implementation capacity.

### 4.7 Limitations

Several limitations require acknowledgement. The organizational policy sample of twelve documents was designed to maximize variation across sectors, sizes and geographic contexts, but it is not statistically representative of South African employers. Reliance on publicly available documents over-represents large organizations with established online policy infrastructure and under-represents small and medium-sized enterprises. Private sector policies dominate the sample (seven of twelve), and the single non-profit policy provides limited insight into civil society approaches. The geographic spread across five provinces does not support conclusions about workplace mental health policy in rural and remote areas, and no provincial comparisons are drawn.

The analysis is restricted to the textual content of the primary statutes and the sampled policies. Labour court decisions, CCMA awards, judicial interpretation, collective agreements, labour inspection records and implementation outcomes were not systematically examined. The conclusions therefore concern discursive and regulatory constructions rather than the law as interpreted and applied in practice. Subordinate legislation, including regulations, codes and administrative guidance issued under each Act, was not subjected to the same systematic review as the primary statutes; where such instruments are referred to, they serve as context rather than corpus. Where enforcement capacity is discussed, the interpretation draws on cited contextual literature rather than treating textual silence as direct evidence of implementation failure (14). The absence of primary data from employees or employers limits understanding of how the identified discursive patterns are experienced and navigated in working life. Because PSC was not measured, the relationships proposed between the analysed discourse and PSC remain interpretive. Future research should combine institutional discourse analysis with organizational and individual-level data, including qualitative interviews, participatory designs and direct PSC measurement, to examine the pathways this study can only propose.

## 5 Recommendations

The recommendations that follow derive from the discursive patterns identified in the Results and are organised at five levels. They are the study's recommendations; the term reform is used only for the legislative and institutional changes being advocated. Their common thread is the movement of psychological health from discretionary benefit towards governed obligation.

### 5.1 Legal reform

The OHSA should be amended to include explicit psychosocial risk provisions. The general duty in section 8 should be supported by regulations requiring employers to identify, assess and mitigate psychosocial hazards, parallel to the Act's existing physical risk management framework, with ISO 45003 available as a practical reference standard (41) and the role of health and safety representatives extended to psychosocial risk monitoring. The EEA and its Code of Good Practice on the Employment of Persons with Disabilities should be expanded to address psychosocial disability explicitly, including operational guidance for work-related and episodic conditions, attention to the barriers associated with disclosure-triggered accommodation, and the specification of psychosocial disability within employment equity planning and reporting. The LRA should support psychosocial protection through clearer guidance on fair treatment following mental health disclosure or incapacity, the consideration of reasonable alternatives to dismissal and the inclusion of psychosocial working conditions within collective bargaining and consultation. The BCEA's working-time and rest provisions should be supported by guidance recognising their role in controlling fatigue, excessive demands and inadequate recovery. Primary responsibility for the identification and control of psychosocial hazards should remain within the occupational health and safety framework (25, 26, 42).

### 5.2 Regulatory and enforcement reform

The Department of Employment and Labour should invest in psychosocial risk training for labour inspectors and issue regulatory guidance or a code of good practice on workplace psychological health. The Compensation Fund should review and broaden the mechanisms through which work-related psychological conditions are recognised and compensated. Existing provision for post-traumatic stress disorder offers an important but relatively narrow entry point, particularly where compensability depends on an identifiable traumatic incident, and greater attention is required to psychological harm associated with cumulative psychosocial exposure. The implementation of the rehabilitation, reintegration and return-to-work framework should also be monitored to ensure that employees with work-related psychological conditions receive appropriate vocational rehabilitation, workplace adjustment and sustained return-to-work support. These measures require interdepartmental coordination: the Department of Health should contribute clinical expertise through the National Mental Health Policy Framework, and legislative amendments should pass through NEDLAC to secure social dialogue legitimacy. Strengthened provisions without strengthened enforcement infrastructure would reproduce the symbolic pattern identified in the policy corpus (14, 48).

### 5.3 Organizational policy reform

Organizational mental health policies should specify measurable responsibilities, prevention procedures, and monitoring and accountability mechanisms, including indicators, responsible actors and reporting intervals. Policies should provide for employee participation in their development and review, multilingual communication, and culturally responsive provisions that recognise the diverse frameworks through which employees understand and seek support for psychological distress (7, 27, 34).

### 5.4 Human resource practice reform

HR practitioners need not await legislative change. Proactive psychosocial risk assessment is available now: the OHSA does not require such assessments, but it does not preclude them, and the section 8 general duty provides an organizational basis for their adoption. Established psychosocial risk-management frameworks and assessment resources include PRIMA-EF, the Health and Safety Executive's Management Standards approach and associated Indicator Tool, and the Copenhagen Psychosocial Questionnaire. Organizations should select, validate and adapt tools according to workforce characteristics, legal context, technical capacity and applicable use or licensing conditions (56, 59, 60). EAP provision should be repositioned from primary intervention to supplementary support within a prevention-oriented model, integrating managerial early-warning training, team-level psychosocial monitoring and occupational health services (1, 46). Line manager mental health literacy training addresses the primary interface between employees and organizational support (34). Culturally competent policy co-design, involving employees from diverse cultural, linguistic and occupational backgrounds, responds directly to the cultural invisibility identified in the corpus (7, 27). Internal accountability mechanisms, including annual psychosocial risk reporting and well-being metrics in management performance frameworks, address the accountability gap identified in the policy sample (48).

### 5.5 Collective bargaining and organised labour reform

Psychosocial safety should be recognised as a legitimate subject of collective bargaining. The LRA permits parties to negotiate beyond statutory minimums, and psychosocial risk assessment, reasonable accommodation and culturally competent provisions can be incorporated into collective agreements under the existing architecture. Model psychosocial health clauses should be developed for sectoral agreements, trade unions should be involved in the development and review of organizational mental health policies, and the capacity of health and safety committees to raise, discuss and refer psychosocial risks (OHSA, ss 19–20) should be strengthened. Collective monitoring of organizational commitments would supply the accountability mechanism that the analysed policies lack and would begin to restore the collective voice whose absence this study documents (35–38).

## 6 Conclusion

This study presented a qualitative documentary analysis of four South African employment statutes and twelve organizational mental health policies, using a Fairclough-informed critical discourse analysis structured by three regulatory dimensions: psychosocial hazard prevention, reasonable accommodation for psychosocial disability, and protection from mental health-related discrimination.

South African labour legislation and organizational policies provide fragmented and largely reactive entry points for employee mental health protection. The analysed discourse appears to position psychological health as a matter of incapacity, individual support and employer discretion more often than as a collective and preventive governance obligation. Cultural diversity is largely invisible in the texts, comprehensive accountability mechanisms were largely absent from the policy corpus, and organised labour is a missing governance actor in a domain it has historically shaped. These patterns may constrain the regulatory and organizational conditions that support a strong psychosocial safety climate, although PSC and employee outcomes were not directly measured, and the study identifies discursive constructions rather than legal effectiveness in practice.

The study's recommendations address legal, regulatory, organizational, human resource and collective bargaining levels. They are not exhaustive, but they identify the structural foundations on which incremental improvement can build. Reframing employee mental health from a discretionary benefit towards an explicit governance responsibility may strengthen constitutional alignment, support the organizational conditions associated with PSC and advance more dignified and psychologically safe employment.

## Appendix A. Anonymised Corpus Profile and Analytical Policy Orientations

Appendix A reports the organizational policy corpus at group level. Formal policy titles and formal document-type labels are not reproduced. The analytical policy orientation column describes the substantive orientation evidenced in the retained coding record and manuscript. It should not be read as the organization's official policy title or formal classification. Short expressions in quotation marks are the exact lexical indicators retained in the analytical record. Geographic information is descriptive and does not support provincial comparison.

**Table A1:** Grouped anonymised profile of the 12 organizational mental health policies included in the critical discourse analysis, reporting sector, number of documents, organizational size, geographic base, national footprint, year range, analytical policy orientation and the exact lexical indicators retained in the analytical record.

Group	Sector/industry	n	Size	Primary base	National footprint	Year(s)	Analytical policy orientation	Retained lexical indicators
A1	Private: mining	2	Large	Gauteng	Yes	2022–2023	Occupational health, fitness-for-duty and operational-risk orientation	“fitness for duty”; “risk management”
A2	Private: finance	2	Large	Gauteng; Western Cape	Mixed	2022–2023	Employee wellness, resilience and performance-support orientation	“resilience”; “performance optimisation”
A3	Private: retail	1	Large	Gauteng	No	2021	Employee mental-health and wellness-support orientation	No document-specific lexical indicator retained in the manuscript
A4	Private: manufacturing	1	Medium	Eastern Cape	No	2023	Integrated employee-wellness and EAP-related orientation	No document-specific lexical indicator retained in the manuscript
A5	Private: technology	1	Medium	KwaZulu-Natal	No	2024	Employee well-being benefits and individual-support orientation	“wellness days”; “mental health stipends”
A6	Public sector: national department, provincial administrations and state-owned enterprise	4	Large and medium	Pretoria; Western Cape; Mpumalanga; KwaZulu-Natal	Mixed	2022–2023	Occupational health, incapacity-management, return-to-work and employee-wellness orientation	“incapacity management”; “return-to-work”
A7	Non-profit: health sector	1	Small	National, multi-province	Yes	2024	Staff well-being, burnout-prevention and compassionate-support orientation	“burnout prevention”; “compassionate leave”; “staff well-being”

“National footprint” indicates operation across all nine South African provinces; “Mixed” indicates that the group contains organizations with and without a national footprint. Year refers to the most recent date of policy publication or revision at the time of retrieval.
